# Exploring the developmental mechanisms underlying Wolf-Hirschhorn Syndrome: Evidence for defects in neural crest cell migration

**DOI:** 10.1016/j.ydbio.2016.10.012

**Published:** 2016-10-21

**Authors:** Erin L. Rutherford, Laura Anne Lowery

**Affiliations:** Boston College, Department of Biology, 140 Commonwealth Avenue, Chestnut Hill, MA 02467, United States

**Keywords:** Wolf-Hirschhorn Syndrome, Neural crest, Cell migration, Embryonic development

## Abstract

Wolf-Hirschhorn Syndrome (WHS) is a neurodevelopmental disorder characterized by mental retardation, craniofacial malformation, and defects in skeletal and heart development. The syndrome is associated with irregularities on the short arm of chromosome 4, including deletions of varying sizes and microduplications. Many of these genotypic aberrations in humans have been correlated with the classic WHS phenotype, and animal models have provided a context for mapping these genetic irregularities to specific phenotypes; however, there remains a significant knowledge gap concerning the cell biological mechanisms underlying these phenotypes. This review summarizes literature that has made recent contributions to this topic, drawing from the vast body of knowledge detailing the genetic particularities of the disorder and the more limited pool of information on its cell biology. Finally, we propose a novel characterization for WHS as a pathophysiology owing in part to defects in neural crest cell motility and migration during development.

## 1. Introduction

Wolf-Hirschhorn Syndrome (WHS) is a developmental disorder characterized by intellectual disability, craniofacial abnormalities, heart defects, skeletal defects, urogenital defects, and seizures (Battaglia et al., 2015; Cooper and Hirschhorn, 1961; Wolf et al., 1965). The most obvious and common clinical marker of WHS is the “Greek warrior helmet” appearance, caused by an abnormally wide nasal bridge attaching the nose to the forehead. WHS patients also exhibit a high forehead, drastic eyebrow arches, widely spaced eyes, a short philtrum, and micrognathia (undersized jaw). The vast majority of patients are microcephalic and have ears marked by abnormal positioning on the head and underdeveloped cartilage. Defects of the eye and optic nerve also occur in approximately 40% of patients. Many facial phenotypes can be classified as midline defects, including the common cleft palate. An overarching growth delay is also notable in WHS patients; the onset is prenatal in most and continues to manifest throughout early development, as patients’ stature is short and weight gain is slow (Battaglia et al., 2015). As the pathways responsible for brain development and craniofacial morphogenesis are linked (Brault et al., 2001; Marcucio et al., 2005), it is not surprising that a majority of WHS patients also exhibit mental retardation (Bergemann et al., 2005).

Each patient presents with a unique combination of WHS characteristics, with varying severity. Such clinical variability is the first reason that it has proven difficult to conduct research into the disorder’s underlying pathology. This review will focus on a second reason: WHS manifests at the genomic, epigenomic, and cell biological levels, and there is a lack of mechanistic knowledge regarding the exact effectors downstream of certain genetic mutations. Consequently, the field still lacks insights into appropriate initial questions to jumpstart investigations into the cell biological functions of WHS-related proteins. However, several players in the processes underlying the disorder have previously been described —for the most part, these are proteins which have established roles in routine epigenetic modification, normal cellular metabolism, or in signaling pathways integral to proper development. From a few early studies, it has become clear that some of these epigenetic modifications and signaling events are connected to cell motility-related processes. Furthermore, the characteristic facial (and cardiac) phenotypes of WHS call to mind the neural crest, as most vertebrate facial features are derived from or influenced by the cranial subset of this multipotent stem cell population (Bronner and LeDouarin, 2012). Neural crest cells are born along the embryonic neural tube, and migrate long distances to reach their destinations, where they differentiate and contribute to structures such as peripheral nerves, jaw structures, facial cartilage, elements of the heart, and pigmented epidermal tissue (Bronner and LeDouarin, 2012). Considering both the morphological data detailing the WHS phenotype and recent cell biological studies exploring WHS candidate proteins, neural crest cell motility and migration are a promising avenue for investigation into WHS at the cell biological level.

**Fig. 1:**
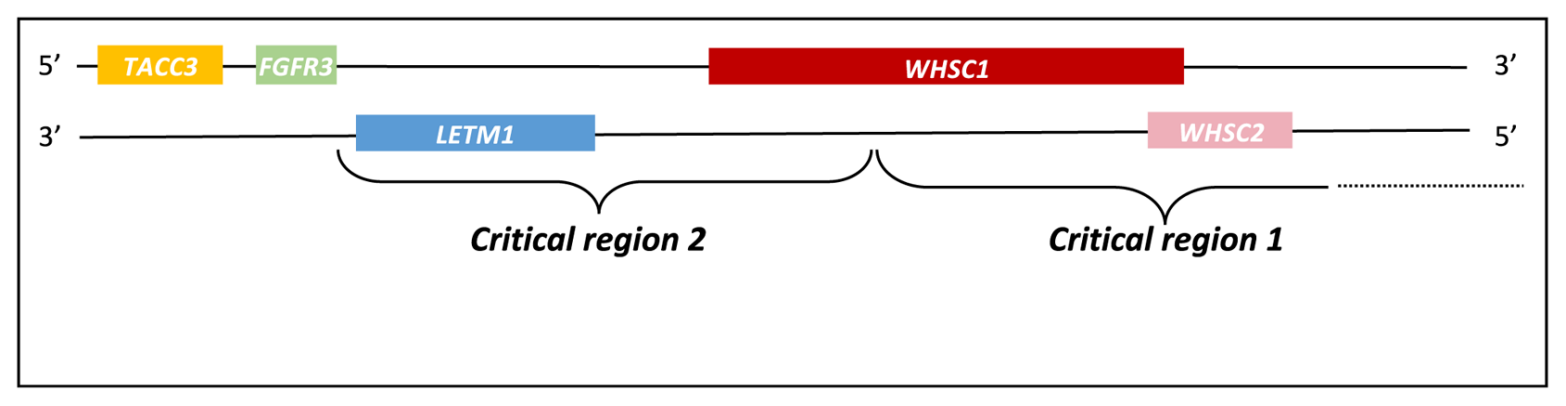
Chromosome 4p16.3 and WHS candidate genes. Genes are depicted in their order from left to right, telomeric to centromeric orientation. Sizes are proportional to the length of the gene in kilobases. Plus or minus strand orientation is also represented. Brackets represent the lengths of the two WHS critical regions. As dotted line indicates, figure scaling is such that critical region one is not represented in its entirety on the page.

Following Hirschhorn’s initial characterization of a clinical phenotype in 1961, the major catalyst for research into WHS has been the definition of a critical genomic region (“critical region 1”), consisting of 165 kilobases on the short arm of chromosome four (the 4p region), which is deleted in traditional cases of the disorder (Wright et al., 1997; Fig. 1). The only characterized candidate genes that fall within this region are Wolf-Hirschhorn candidate 1 (*WHSC1*), referred to elsewhere as a multiple myeloma SET (MMSET) or nuclear receptor SET domain (NSD) protein, and *WHSC2*, which is also known as a negative elongation factor (NELF) (Wright et al., 1999). Despite lying entirely within the WHS critical region, *WHSC2* has not emerged as one of the primary contributors to the WHS phenotype. While this may be due to a deficit in *WHSC2*-based studies, it also results from the identification of several patients with deletions telomeric to the critical region. As *WHSC2* is left unaffected in these cases, they have called into question the gene’s clinical relevance (Bergemann et al., 2005; Cyr et al., 2011; Engbers et al., 2009; Zollino et al., 2014).

Deletions in the 4p region in most WHS patients encompass areas extending beyond the critical region, thus affecting several flanking genes including a transforming acidic coiled-coil gene (*TACC3),* a sequence encoding a fibroblast growth factor receptor product *(FGFR3)*, and the gene for a leucine zipper and EF-hand containing transmembrane protein *(LETM1),* as listed in their sequential order with *TACC3* most telomeric (Zollino et al., 2003). More recent genomic characterizations have revealed synteny with genes found on chromosome 8p11.2 (Stec et al., 2001). It is clear that the *FGFR-LETM-WHSC1* ordering is ancient, as it is conserved from fish to humans. Moreover, there are speculations as to whether this conserved region is larger, including *TACC* and *NELF/WHSC2,* and whether this particular grouping indicates some functional relationship (Stec et al., 2001). This review will explore the latter possibility by focusing in on the WHSC1 protein encoded on chromosome 4p16.3 (where WHS mutations typically occur), as well as on FGFR3, LETM1, and TACC3, as these are presently the most promising candidates that may demonstrate a novel functional relationship based on neural crest cell motility in WHS (Table 1). Clinical variation in 4p deletion size has highlighted other genes (*SLBP, CTBP1, CPLX1, PIGG, FGFRL1*) as potential WHS contributors (Battaglia et al., 2015); however, a smaller group are discussed in this review to best articulate potential cell biological intersections between specific candidate proteins with putative roles neural crest cell migration. Importantly, hemizygous deletions in the 4p region have proven sufficient for the onset of traditional WHS (Battaglia et al., 2015). In such a conserved and vital region, it is worthwhile to consider each candidate protein alongside its genomic neighbors; this inclusive approach may illuminate ways in which aberrant regulation of several WHS candidate proteins may converge to impact cell migration events which are crucial for early morphogenesis and neuronal wiring.

## 2. WHSC1

Of all the candidate genes, *WHSC1* is most often found to be partially or fully monosomic in a majority of clinical cases; however, its absence does not account for the full range of phenotypes associated with the disorder. *WHSC1* is a 90 kb gene, two-thirds of which extend into the telomeric end of the 165 kb Wolf-Hirschhorn Syndrome Critical Region. It contains 25 exons and is subject to complex alternative splicing (Stec et al., 1998). Northern blot analysis of expression patterns in human and mouse embryonic and fetal tissues are highly variable and suggest that many different tissue-specific transcripts exist, with 9 kb and 6 kb transcripts consistently appearing across tissue types. Between exons 4 (where the translational start site is located) and 25, there is a 4095 base pair open reading frame (ORF) from which the WHSC1-encoding mRNA is transcribed (Stec et al., 1998). *In situ* hybridization in mouse revealed a specific pattern of *WHSC1* expression in the developing nervous system at day 10.5, and expression in brain, ganglia, neural tube, jaw, face, intestinal and lung epithelium, liver, adrenal, and urogenital region at day 13.5. Such an expression pattern is significant given that many of these—notably the brain, jaw, face, and urogenital structures—are affected in the classical WHS phenotype (Stec et al., 1998), and given that the formation of structures of the face and jaw, as well as certain glial cell populations in the brain, depend on neural crest cell migration (Bronner and LeDouarin, 2012). However, WHSC1 function has not yet been explicitly linked to neural crest cell migration.

### 2.1. WHSC1 as a histone modifier during development

Although there is a reigning generalization in the field that abnormal *WHSC1* dosage is responsible for many of the defining phenotypes of WHS (Bergemann et al., 2005), the question of how it causes such phenotypes remains unanswered. The protein product of this gene is also called MMSET or NSD2, as it is characterized by several domains with known importance to development, including an HMG box, a PHD-type zinc finger domain, and a SET domain, all involved in regulation of transcription during development (Stec et al., 1998). The SET domain possesses methyltransferase capabilities and is common to all but one member of the histone lysine methyltransferase family (Marango et al., 2008; Völkel and Angrand, 2007). Experiments using recombinant NSD proteins have shown that these histone lysine methyltransferases can specifically dimethylate lysine 36 of histone H3 (H3K36) given a nucleosome substrate (Li et al., 2009; Venkatesh and Workman, 2013). Additionally, methylation of histone H3 following the transcription of genes associated with this histone (termed co-transcriptional methylation) correlates with the recruitment of histone deacetylase complexes (Venkatesh and Workman, 2013). This is significant because deacetylation and methylation markers at H3—which together suppress untimely transcript initiation—are involved in ensuring the appropriate timing of gene expression. Thus, these marks are especially relevant in early development. Nimura et al. implicate interactions between WHSC1 and cell-type specific transcription factors (TFs) as being responsible for the protein’s specific methylation pattern at certain genomic loci: immunoaffinity purification of murine Whsc1-associated proteins—followed by mass spectrometry and pull-down assays—revealed interactions with developmental TFs Sall1, Sall4, and Nanog in ES cells, and Nkx2-5 in embryonic hearts (Nimura et al., 2009). The interactions between WHSC1 and these proteins may prevent inappropriate transcription, as histone deacetylase 1 also interacts with Whsc1-TF complexes and could confer transcriptional repression via the removal of the acetyl mark. This study illuminates one of the many possible functions of WHSC1 in development, and addresses just a few of its possible interactors (Nimura et al., 2009). As indicated by RT-PCR analysis in zebrafish, the mRNA transcript for the homolog *DrWhsc1* is present in most adult tissue types (Yamada-Okabe et al., 2010); given its prevalence in both mature and immature cell types, it seems probable that developmentally-regulated transcription factor interactions are just one of many roles of WHSC1 within the cell.

### 2.2. Involvement of WHSC1 in Wnt signaling

**Fig. 2:**
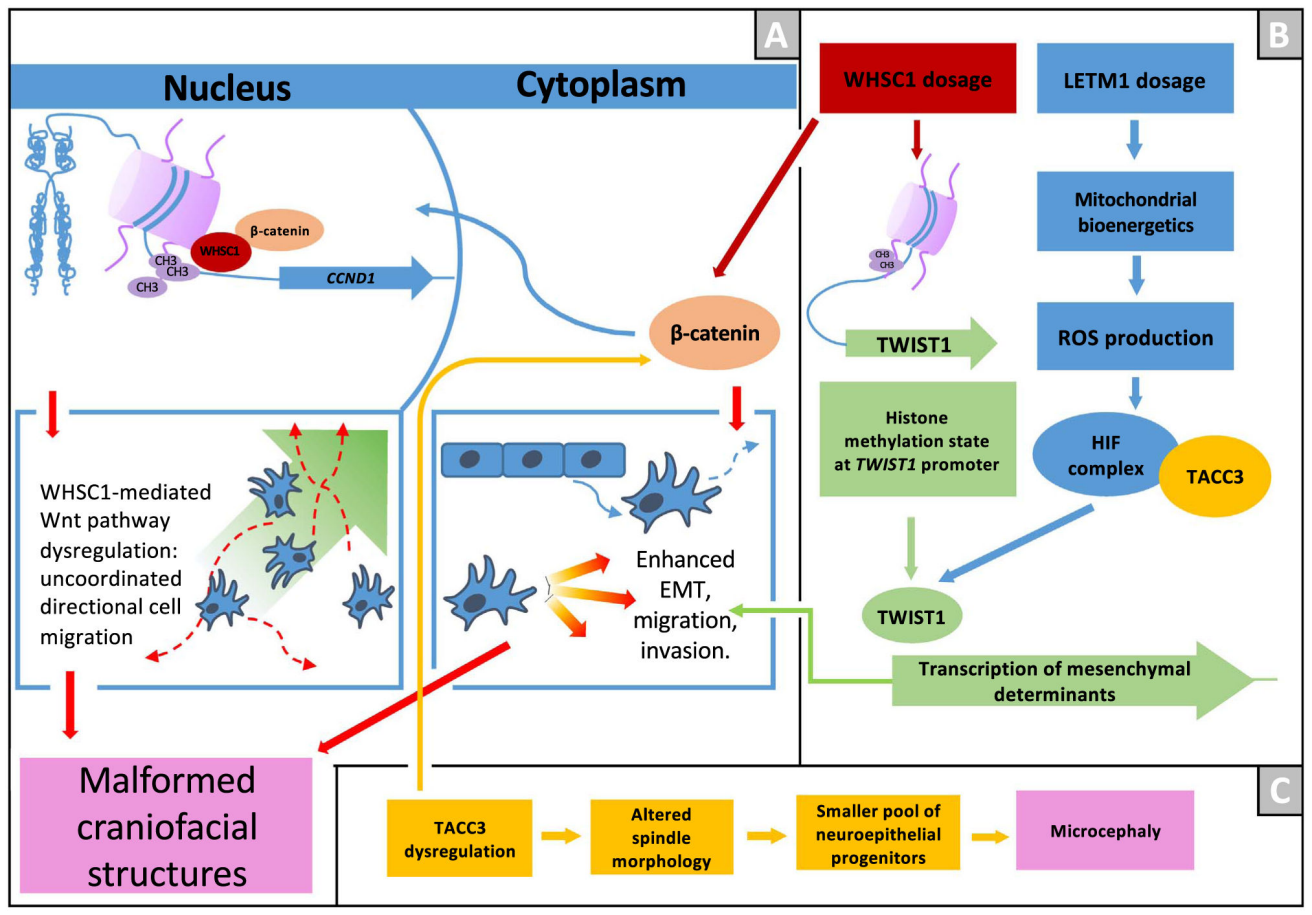
Potential pathways by which expression levels of WHS candidate proteins may contribute to disorder phenotypes via dysregulated cell migration. (A) WHSC1 and beta-catenin interact in the nucleus, where WHSC1 alters histone methylation surrounding *CCND1*, a beta-catenin target gene. It has been suggested that WHSC1 dosage may influence subcellular localization of beta-catenin, thus changing signaling events that would normally occur downstream of Wnt. This in turn may affect the tightlycontrolled timing of EMT, especially if levels of cytoplasm/cell surface-associated beta-catenin are altered sufficiently to affect epithelial junctions. Additionally, signaling downstream of Wnt has proven essential for the coordinated, directional motility of migratory neural crest cells, thus aberrant signaling may affect cell migration patterns. (B) WHSC1 controls histone methylation state at the *TWIST1* promoter, thus abnormally low WHSC1 expression levels may influence the ability of TWIST to aid in transcription of mesenchymal determinants when necessary. Additionally, altered bioenergetics due to LETM1 deficiency may affect the activation of the hypoxia inducible factor complex, which lies upstream of TWIST activation. (C) TACC3 protein levels are known to affect spindle morphology, and may contribute to microcephaly by causing abnormal spindles which impair the regulated transition from symmetric to asymmetric cell divisions, decreasing the quantity of neuroepithelial progenitors. TACC3 has further roles in regulating cell motility, as its over-expression has been shown to increase beta-catenin targeting to the nucleus, and to increase the invasive potential of migrating mesenchymal cells (shown in A). Moreover, it is a co-factor to the hypoxia inducible factor complex; this highlights TWIST activation as another regulatory point at which *TACC3* dosage could affect the epithelial/mesenchymal fate of pre-migratory cells.

Because WHSC1 is a chromatin modifier, an important question regarding its bigger-picture roles relates to which pathways and processes it might affect at the epigenetic level, under normal conditions as well as in a disease state. The work of Toyokawa et al. provides a preliminary answer. Although the field lacks direct evidence of WHSC1 regulation contributing to craniofacial phenotypes through a specific signaling pathway, they have connected WHSC1 dysregulation to Wnt signaling in the context of cancer (Toyokawa et al., 2011). This group observed upregulation of WHSC1 at the protein level, in addition to elevated levels of its transcribed RNA, in human cancer cell lines. They propose a model in which WHSC1 interaction with beta-catenin is responsible for certain genomic instances of H3K36me3. Specifically, Toyokawa et al. note that H3K36me3 is enriched at the transcriptional start site of *CCND1*, one indirect target of the beta-catenin-Tcf-4 complex and a downstream effector of Wnt signaling (Sansom et al., 2005; Toyokawa et al., 2011; Fig. 2). Further evidence supporting a role for Wnt dysregulation in WHS was gathered from immunoprecipitation-mass spectrometry, which identified WHSC1 interactors IQGAP1 and beta-catenin (Toyokawa et al., 2011) both of which are involved in Wnt signaling as well as cadherin-mediated cell-cell adhesion and cell migration (Goto et al., 2013; Noritake et al., 2005). It was also reported that nucleus/cytoplasm fractionation assays pinpointed the nucleus as the place of interaction between WHSC1 and beta-catenin (Toyokawa et al., 2011; Fig. 2A). Toyokawa et al. note the possibility that WHSC1 dosage may impact nuclear levels of beta-catenin, in turn affecting canonical Wnt signaling through transcriptional regulation.

The importance of properly-controlled Wnt signaling in developmental cell migration highlights the need for a clearer vision of how *WHSC1* dosage impacts this signaling pathway. Particularly significant in migrating neural crest cells are non-canonical Wnt signaling events, which affect Rho/Rac regulation of the actin cytoskeleton, and are a driving force for the generation of planar cell polarity in the migratory neural crest (Mayor and Theveneau, 2014). Planar cell polarity (PCP) signaling depends on the asymmetric localization of molecules, such as non-canonical Wnt11 ligand, the Wnt receptor Frizzled, and the signaling protein Disheveled, to contacts between neighboring cells (Mayor and Theveneau, 2014). In *Xenopus*, Wnt11 is expressed adjacent and lateral to the early cranial neural crest, and plays an essential role in neural crest cell migration through activation of the PCP pathway (De Calisto et al., 2005). As a whole, PCP signaling allows cell protrusions to form towards open areas within migrating neural crest cell streams, and towards the boundaries of cell populations expressing attractive cues; likewise, it restricts their formation between neighboring cells which have made contact (thus initiating contact inhibition of locomotion), and between cells and surrounding tissues providing repellent signals (Mayor and Theveneau, 2014). As non-canonical Wnt/PCP signaling is necessary for establishing proper directionality of neural crest cell migration (Carmona-Fontaine et al., 2008; De Calisto et al., 2005), its disruption could result in faulty patterns with eventual effects manifesting as craniofacial abnormalities (Fig. 2A).

Further experiments looking specifically at facial morphogenesis have shown that Wnt proteins are powerful regulators of regional facial patterning, and that blocking their activity causes midfacial malformation in chicks (Brugmann et al., 2007). Although it has become clear that canonical Wnt/beta-catenin signaling is vital for neural crest induction (García-Castro et al., 2002; Leung et al., 2016; Wang et al., 2010), the finding that these same Wnt pathways are active in specific populations of delaminating and migrating murine cranial neural crest cells indicates an ongoing role in mammalian facial patterning beyond cell fate specification (Brugmann et al., 2007). In sum, the importance of spatiotemporally-controlled Wnt signaling in embryonic development, and the newly-elucidated connection to WHSC1, qualify both canonical and non-canonical Wnt dysregulation in the neural crest as possible contributors to the orofacial and midline defects observed in the classic WHS phenotype.

### 2.3. WHSC1 as an epigenetic regulator of Twist expression

Further study of WHSC1 in cancer cell lines strengthens the link between WHS and aberrant cell migration, as the WHSC1 methyl-transferase activity has been shown to exert epigenetic control over the expression of Twist family proteins, transcription factors that regulates epithelial-to-mesenchymal transition (EMT) in normal development, as well as in cancer cells (Ezponda et al., 2013; Kuo et al., 2013; Yang et al., 2004; Fig. 2B). As shown by microarray and confirmed by RT-PCR and immunoblot at the RNA and protein levels, *WHSC1* is among a small pool of genes which are up-regulated after treatment with resistin, an EMT-inducing paracrine factor secreted by lung tumor-associated dendritic cells in humans (Kuo et al., 2013). In this system, abnormally high WHSC1 dosage causes dimethylation of histone H3K36 and decreased trimethylation of H3K27, an epigenetic combination which in turn enhances the expression of the transcription factor Twist. As a master regulator of EMT, Twist drives a change in cellular identity by initiating large-scale changes in gene expression; in the case of these WHSC1 over-expressing cell lines, overactive Twist likely explains the enhanced migration and invasion behaviors observed by Kuo et al. Importantly, there was no change in proliferation levels, affirming that altered cell migration is the major WHSC1 over-expression phenotype of developmental relevance (Kuo et al., 2013). Twist hyperactivity has also been shown in prostate cancer as a result of WHSC1 over-expression: WHSC1 binds to the *TWIST1* locus to activate its expression by means of increased H3K36 dimethylation. Thus, when TWIST1 is knocked down in WHSC1-over-expressing RWPE-1 cells, EMT and invasion are prevented (Ezponda et al., 2013). Considering these data, there is strong support that aspects of the WHS phenotype result from aberrantly regulated EMT and, subsequently, cell migration.

### 2.4. Neural crest cell migration is regulated by histone H3 methyltransferases

Neural crest cells rely on continuous epigenetic modifications to drive changes in gene expression during their migration (Hu et al., 2014). WHSC1L1—the paralog of WHSC1 found on chromosome 8—is a major NSD-family histone methyltransferase thought to facilitate such changes at H3K36 of the neural crest cell transcription factor *Sox10* (Jacques-Fricke and Gammill, 2014). Electroporating chick embryos before neural crest cell migration with a dominant negative construct lacking the methylating SET domain resulted in faulty migration: cells began to travel away from the neural tube but stopped short of their destination points (Jacques-Fricke and Gammill, 2014). Additionally, this dominant negative construct disrupted specification, preventing the expression of neural crest-characterizing transcripts *Msx1, FoxD3, Sox9*, *Sox10,* and *Snail2,* as visualized by in situ hybridization. It is unclear exactly how NSD-family proteins regulate neural crest identity and migration patterns. Despite the evidence that WHSC1L1 is a major methylating agent during migration, it only directly methylates at the *Sox10* genomic region (Jacques-Fricke and Gammill, 2014); this leaves open possible roles for other NSD family proteins such as WHSC1 in driving as-of-yet unexplored epigenetic modifications during neural crest cell specification and migration. Thus, chromosome 4p deletions characteristic of WHS may indeed impact neural crest development, especially considering the similar structures and functions conserved from *WHSC1L1* and *WHSC1*, the 4p gene from which it arose by way of a duplication event (Stec et al., 2001).

### 2.5. Future directions: mechanistically interrogating the role of WHSC1 in cell motility

What still remains uncertain are the exact mechanisms through which WHSC1 impacts cell motility. One study in a multiple myeloma cell line marked by a genetic translocation causing WHSC1 up-regulation showed that WHSC1 knockdown led to cell cycle arrest, reduced tumorigenicity, and inhibited extracellular matrix adherence (Lauring et al., 2008). Thus, regulation of cell adhesion is one possible mechanism. Moreover, siRNA-mediated knockdown of WHSC1 decreased expression levels of genes involved in cell adhesion, namely DSG2, a component of desmosomes also known to impact proliferation and signaling, and ADAM9, a metalloproteinase which interacts with αvβ5 integrin and promotes substrate adhesion (Brito et al., 2009). Further study will be needed to corroborate the link between WHSC1 dysregulation and adhesion-mediated defects in motility.

## 3. LETM1

*LETM1* is a gene located less than 80 kb distal to the minimal WHS critical region between *WHSC1* and *FGFR3*, and it is deleted in most WHS patients (Endele et al., 1999). After initial cloning and characterization of the gene, a putative protein was inferred from its sequence, and it was predicted to have several coiled-coil regions and a leucine zipper as its defining structural features; its sequence homology grouped it with the EF-hand calcium-binding protein family (Endele et al., 1999). Subsequent work examined its conserved expression and localization from yeast to vertebrates and revealed that, in mouse embryonic fibroblasts and in yeast, LETM1 and its ortholog, YOL027, encode integral proteins found in the inner mitochondrial membrane (Nowikovsky et al., 2004). Conservation of protein function was also examined between human and yeast homologs; *Yol027*-deficient yeast could be rescued by expression of human *LETM1*, suggesting that human LETM1 is also targeted to yeast mitochondria and restores the function of its missing ortholog (Nowikovsky et al., 2004).

### 3.1. *LETM1* as a component of a new WHS critical region

In keeping with the trajectory of WHS research thus far, recent investigation into LETM1 has attempted to reconcile questions of protein function with an ever-shifting understanding of the disorder’s genetic roots, often gained from phenotypic and genotypic analysis of a small sample of human patients. The expansion of the WHS critical region is one important instance: a second, larger WHS critical region was defined by Zollino et al. in 2003. It falls within a 300–600-kb interval in 4p16.3, between loci D4S3327 and D4S98-D4S168, and includes *LETM1* (Zollino et al., 2003). Such an expansion accommodates not only the phenotype-genotype correlations observed in the patients described by Zollino et al.—who exhibited WHS hallmarks such as seizures, microcephaly, abnormal facial appearance, and growth delay, despite having an intact WHS critical region—but also a patient documented in Rauch et al., whose critical region exhibited a 191.5-kb deletion which left *LETM1* unaffected (Rauch et al., 2001). This patient’s minor growth and learning impairment, distinct facial morphology, and marked lack of seizures have led many to postulate that *LETM1* genetic irregularities account for the neuromuscular manifestations of WHS, while other candidate genes in the critical region contribute to additional aspects of the phenotype.

### 3.2. LETM1 deficiency and mitochondrial dysfunction

Addressing the hypothesis that LETM1 dosage affects seizure propensity in WHS patients began with the acquisition of a basic structure-function understanding of the protein: using an EGFP-tagged human LETM1 construct transfected into HEK293 cells, Schlickum et al. corroborated the mitochondrial localization reported by Nowikovsky et al. Moreover, a 167-amino acid deletion at the amino terminus of LETM1 revealed this segment to be the mitochondrial targeting sequence of the protein (Schlickum et al., 2004). The important question of what role LETM1 plays in the inner mitochondrial membrane, and how it may contribute to seizure phenotypes, was pioneered by Jiang et al. (Jiang et al., 2013). Although mitochondrial morphology was similar in primary fibroblast cultures from LETM1 heterozygous knockout mice and WT mice, mitochondria from the heterozygous mutants exhibited altered calcium metabolism and pH compared to those of WT (Jiang et al., 2013). Additionally, LETM1-deficient fibroblasts took up less oxygen than controls in a low-glucose environment, implying a role for LETM1 in the efficiency of glucose oxidation. This notion was supported by tissue-specific analysis of ATP concentrations, which demonstrated a 27% reduction in brain ATP (Jiang et al., 2013). The group subsequently investigated how LETM1 dosage contributes to seizure tendencies in mice, demonstrating seizure scores for LETM1 hemizygous knockout mice to be 1.5-fold higher than that of WT, and their brain ATP concentrations to be reduced by 25% following induced seizures. While the *in vitro* experiments in this study significantly advanced the field’s understanding of the biochemistry of LETM1, these *in vivo* experiments prominently accent the void of cell biological data linking seizure propensity with LETM1-mediated mitochondrial dysfunction.

### 3.3. Potential effects of LETM1 dosage on cellular metabolism

As the downstream effects of cellular calcium and oxygen flux are far-ranging and varied based on cell type, it is prudent to assume that *LETM1* dosage may contribute to the WHS phenotype in ways that are separate from its putative role in seizure-causing depolarization. Recently, Doonan et al. have observed altered mitochondrial calcium influx and eflux, mitochondrial bioenergetics, and metabolic signaling when *LETM1* is silenced in multiple organisms and cell types, including in cells derived from WHS patients; they also report AMPK activation and increased mitochondrial ROS production (Doonan et al., 2014a). While the primary takeaway from this study is that further work is needed in order to associate these metabolic trends with the particulars of the WHS pathophysiology, the identification of such trends is invaluable as a jumping-off point for future studies. Perhaps the group’s most salient observation is the one regarding ROS production, as this phenomenon underlies neurodegeneration in a broad spectrum of diseases, including Alzheimer’s and ALS (Dasuri et al., 2013). The idea that events downstream of ROS production could impact a variety of motile cells just as readily as they affect neuronal networks implies another potential link to the transcription factor Twist (Fig. 2B). Twist is activated by hypoxia-inducible factor (HIF) 1 (Yang and Wu, 2008), and ROS production under hypoxic conditions is necessary and sufficient for HIF activation (Chandel et al., 1998, 2000). Given that LETM1 deficiency impairs oxygen consumption and increases ROS production (Doonan et al., 2014b), a pathological role for HIF complexes upstream of Twist is not out of the question. It is possible that—due to some metabolic irregularity resulting from improper *LETM1* dosage—Twist upregulates mesenchymal phenotypic markers to override normal physiological controls and induce untimely migration events at key moments in development.

## 4. FGFR3

Fibroblast growth factor receptor 3 (FGFR3) is one of the four homologous receptors of the FGFR subgroup, belonging to the receptor tyrosine kinase family. Its activation is associated with cellular processes such as proliferation, migration, cell survival, and wound healing (Eswarakumar et al., 2005). The receptor is activated following dimerization of monomers and subsequent trans-autophosphorylation of the kinase domain, ultimately activating RAS-MAPK, PI3-AKT, and JAK/STAT signaling cascade (Eswarakumar et al., 2005). While roles for FGF receptors in cancer-related processes, such as inappropriate migration and proliferation, have been comprehensively reviewed (Katoh and Nakagama, 2014), less attention has been paid to the cell biological ramifications of FGFR3 absence as it specifically relates to early development.

### 4.1. Effects of FGFR3 deficiency in murine development

**Fig. 3:**
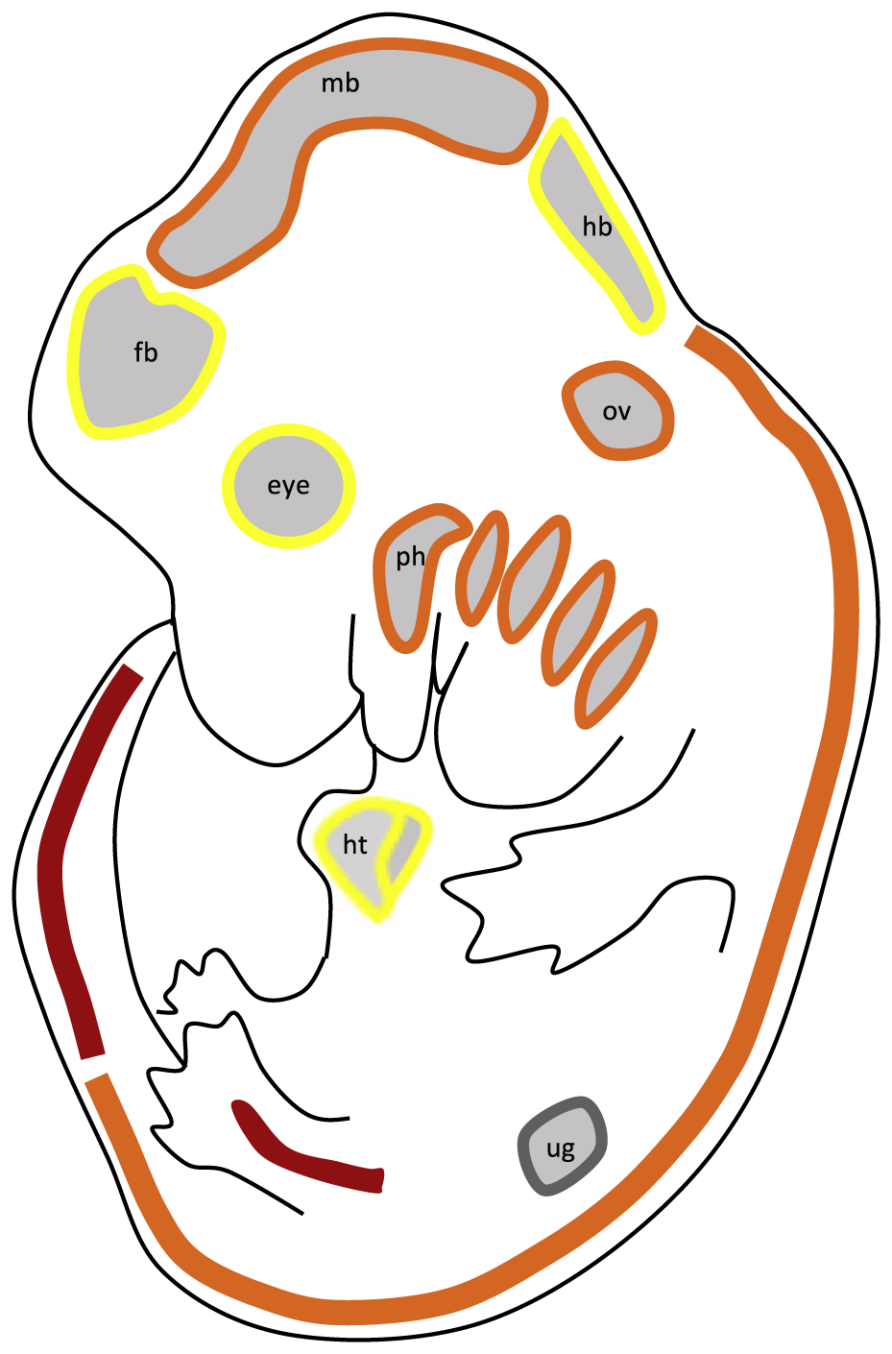
Overlap between developmental structures formed by migrating cells and structures affected in WHS mouse models. Gray areas are murine developmental intermediates that correspond to human structures affected in clinical WHS. Yellow highlights hotspots of cell migration during morphogenesis. Red denotes regions that are malformed in mouse models of WHS. Orange areas highlight the overlap between the two groups. Clinically relevant areas of overlap between cell migration hotspots and malformed structures in murine models include the midbrain (mb), where dopaminergic neurons are affected by Fgfr3 deficiency; the otic vesicle (ov), or early ear consisting of cranial neural crest tissue, which is highlighted here because Fgfr3 deficient adult mice display hearing loss; and the pharyngeal arches (ph), which are populated by migrating streams of cranial neural crest cells and form symmetrical facial structures such as the jaw, affected in TACC3 KO mice. Orange shading along the spine indicates area with high density of migrating spinal neurons, and axial defects in Fgfr3-defficient mice, but no corresponding human WHS phenotype. Clinically relevant structures whose morphogenesis depends on cell migration, but are not affected in any current mouse models, include the forebrain (fb) and hindbrain (hb), where neurites traverse the brain to reach synaptic targets; the eye, where retinal neurons form connections important for sensorineural pathways; and the early heart (ht), towards which cardiac neural crest cells migrate to make important structural contributions. Red shading in the tail and it the skeleton of the limbs indicates mouse-specific axial and appendicular abnormalities caused by Fgfr3 deficiency, not observed in human patients. Gray outlined area marks early urogenital region (ug), impacted in human WHS but not yet affected in any WHS mouse models. Mouse cartoon is representative of day E12.

As FGFR3 is located in the vicinity of the WHS critical region, homozygous null mouse lines have been created for phenotypic assessment, and these have recapitulated some of the skeletal malformation seen in human patients with WHS (Simon and Bergemann, 2008; Fig. 3). Kyphosis (abnormal spine curvature), elongated tails, and malformed femurs are characteristics of homozygous null mice (Colvin et al., 1996; Deng et al., 1996), suggesting that murine *Fgfr3* is involved in inhibiting chondrocyte proliferation (Colvin et al., 1996; Deng et al., 1996; Simon and Bergemann, 2008). Hearing problems and malformation of the ear have also been reported, providing another point of correspondence between human and murine WHS (Colvin et al., 1996; Puligilla et al., 2007; Simon and Bergemann, 2008). A further finding of WHS relevance in mouse relates to the cognitive impairment characteristic of the disorder, as *Fgfr3* (in combination with other FGFRs) has been shown to promote cell survival in the neuroectoderm, as well as the production of dopaminergic neurons in the ventral midbrain (Saarimäki-Vire et al., 2007; Simon and Bergemann, 2008). Loss of this gene due to deletions in the WHS critical region may result in neurodevelopmental delay resulting from inappropriately timed cell death or even a failure to produce certain populations of neurons.

### 4.2. FGFR3 in the context of its growth receptor family

Because of the existence of four FGFR homologs in both mice and humans, it is possible that the activities of FGFR3 in particular vary between human and murine tissues, depending on unknown degrees of functional redundancy. Moreover, while studies in mice have helped to dissect out FGFR-specific phenotypes—something that has not been possible in human patients with deletions that encompass multiple genes of the WHS critical region—the cell biological roots of these phenotypes remain unclear. It seems likely, based on basic principles of skeletal development, that FGFR-related skeletal defects may primarily result from problematic regulation of proliferation; however, the roles of FGFRs in other tissues and cell types are less clear. Interestingly, FGFRs 1 and 3 have recently been implicated in the chemotactic response of cardiac neural crest cells to FGF8 in the pharyngeal ecto-and endoderm in chick embryos, operating upstream of the MAPK/ERK pathway (Sato et al., 2011). Loss of FGFR1 or FGFR3 activity in this context resulted in slower neural crest migration velocities and a tendency for cells to die closer to the neural tube (Sato et al., 2011). These defects due to reduction in FRFR3 function likely have repercussions in cardiac development, which might explain the heart dysfunction characteristic of human WHS. More comprehensive studies on FGFR dose dependence in specific developmental processes will be integral to exploring this possibility.

## 5. TACC3

The transforming acidic coiled-coil protein 3 is yet another example of a protein whose dosage may contribute to the WHS phenotype through several mechanisms, including one related to its role in cell motility and migration. Although *TACC3* need not be affected for a traditional WHS diagnosis to be made, its putative role in facial development and its proximity to the WHS critical region is enough to single it out as a possible culprit in aspects of the pathology. TACC3 is a microtubule associated protein which localizes to the centrosome and mitotic spindle (Gergely et al., 2000; Groisman et al., 2000; Peset and Vernos, 2008). It was recently shown to act as a plus-end tracking protein (+TIP) on the ends of polymerizing microtubules, where it associates with the microtubule polymerase, XMAP215, and affects microtubule dynamics in *Xenopus* embryonic cells (Gutierrez-Caballero et al., 2015; Nwagbara et al., 2014). Consistently, TACC3-depleted HeLa cells have shown destabilized microtubules, mitotic arrest, and improper chromosome arrangement (Gergely et al., 2003; Schneider et al., 2007). The question of how *TACC3* dosage impacts craniofacial development *in vivo* is particularly timely, as it was recently shown that TACC3 mRNA is highly expressed in migratory neural crest cells in the *Xenopus laevis* pharyngeal arches; other TACC family members’ transcripts (TACC1 and TACC2) did not mirror this craniofacial expression pattern (Rutherford et al., 2016). Despite its documented roles in cytoskeletal regulation, the ways in which TACC3 expression levels contribute to gross morphological phenotypes have not yet been described.

### 5.1. TACC3 and WHS genetics

The implication of TACC3 in WHS was most recently validated by a case study in which a patient’s microduplication on chromosome 4p resulted in extra copies of *FGFR3*, *LETM1*, and *TACC3* (Cyr et al., 2011). The duplication notably did not affect *WHSC1* or *WHSC2*. Duplication, instead of deletion, as a cause of WHS has been described elsewhere (Hannes et al., 2010), but as Cyr et al. points out, this earlier-reported duplication did in fact encompass *WHSC1* in addition to other genes such as *TACC3,* and so it seemed unclear which genes within the duplicated region accounted for the observed phenotype. Although it is difficult to draw general conclusions from individual patients, it has been suggested that heightened dosage of *TACC3* may somehow contribute to neurodevelopmental delay (Cyr et al., 2011).

There are currently no mouse models that recapitulate any *TACC3*-encompassing microduplications resulting in WHS-like phenotypes, although there are models presenting evidence of skeletal and craniofacial malformation upon *TACC3* deletion (Piekorz et al., 2002) (Fig. 3). In a study by Piekorz et al., two-thirds of the homozygous null embryonic mice which were not lethally affected by TACC3 knockout exhibited facial clefts, in addition to growth retardation leaving many organs underdeveloped compared to controls (Piekorz et al., 2002). Mice homozygous for hypomorphic mutations have been used to study the effects of TACC3 deficiency in early development, as embryonic lethality is common in homozygous null mice: pups die just after birth and exhibit intrauterine growth retardation, as well as defects in formation of the axial skeleton, while their primary fibroblasts show defective mitosis marked by improper chromosome alignment (Yao et al., 2007). Despite the convincing evidence that TACC3 deficiency correlates with craniofacial malformation in mice, there are limited clinical examples of TACC3 deletions in WHS-presenting human patients. One subject in a clinical study was affected by a 4p16.3 deletion encompassing *WHSC1, LETM1, TACC3, FGFR3*, and thirteen additional transcribed genes (Zollino et al., 2014). This four-year-old boy exhibited facial irregularities and a growth delay characteristic of WHS, although his lack of seizures is puzzling given the previously discussed evidence for *LETM1* deficiency. SNP array analysis for another case, a 2-year old showing developmental delay, revealed a deletion on 4p16.3 which encompassed the first three exons of TACC3 (Engbers et al., 2009). This patient exhibited facial characteristics associated with WHS, although she lacked microcephaly, and showed only mild mental retardation. Interestingly, this patient’s deletion did not affect *WHSC1*, suggesting that other candidate genes including TACC3 may play important roles in facial phenotypes such as the characteristic short philtrum and downturned mouth (Engbers et al., 2009).

### 5.2. Linking TACC3 with spindle morphology and microcephaly

The importance of TACC3 at the mitotic spindle calls to attention a particular phenotype characteristic of WHS: microcephaly, or reduced occipital frontal circumference, is a well-documented outcome of spindle dysregulation (Kerzendorfer et al., 2013; Mahmood et al., 2011; Megraw et al., 2011; Thornton and Woods, 2009; Fig. 2C). The link between microcephaly and spindle pole microtubules is substantiated by the observation that all microcephaly-associated genetic defects identified thus far involve proteins with known or predicted roles in forming or maintaining centrosomes and the spindle apparatus (Kerzendorfer et al., 2013). The influence that spindle regulation has on determination of cerebral size is perhaps most clear in the example of ASPM, or abnormal spindle-like microcephaly-associated protein. Knockdown of ASPM by RNA interference in mouse neuroepithelial cells results in skewed spindle pole orientation, preventing one daughter cell from inheriting apical membrane and ultimately preventing the pool of neuroepithelial progenitors from expanding; this reduced progenitor pool size could very well be an early developmental determinant of microcephaly (Fish et al., 2006). As Kerzendorfer et al. point out, progenitor cells’ transition from symmetric to asymmetric division is tightly regulated, so that differentiation and subsequent migration to the appropriate tissue occur normally (Kerzendorfer et al., 2013). Going forward, it will be constructive to examine whether TACC3 and other plus-end tracking proteins (+TIPs) at the mitotic spindle are capable of altering its morphology such that cell fate and migration are impacted, and whether changes in these processes contribute to the defects seen in WHS.

### 5.3. TACC3 involvement in epithelial-to-mesenchymal transition

Recent work has suggested a more direct role for TACC3 in cell migration, similar to some of its WHS-related neighbors encoded on chromosome 4p. Most strikingly, TACC3-over-expressing HeLa cells exhibited up-regulation of mesenchymal markers and corresponding down-regulation of epithelial ones, as well as a distinctly elongated and spindle-like morphology characteristic of EMT (Ha et al., 2013). Additional phenotypes included enhanced proliferation and growth on agar, enhanced migration as determined by transwell assays, and enhanced invasion as shown through a matrigel invasion assay; the same trends were reported for TACC3-over-expressing HEK293 cells and U2OS cells (Ha et al., 2013). TACC3 was also found to be linked with the activity of beta-catenin: reporter assays demonstrated enhanced beta-catenin activity in TACC3-over-expressing cells, and Western blot analysis strengthened this finding by revealing heightened expression (Ha et al., 2013). Immunofluorescence experiments performed by Ha et al. illuminate a possible connection to WHSC1: TACC3 over-expression results in increased nuclear localization of beta-catenin compared to controls (Fig. 2A), and the nucleus is where WHSC1-beta-catenin association is reported to be most prevalent (Toyokawa et al., 2011). Taken together with the mounting evidence detailing beta-catenin’s relationship to EMT (Kim et al., 2002), its heightened nuclear localization following TACC3 over-expression highlights a possible regulatory role for TACC3 upstream of the WHSC1-beta-catenin complex.

### 5.4. Future directions for investigating the role of TACC3 in WHS

Looking ahead, it will be worthwhile to consider alternative ways in which TACC3 could contribute to WHS pathophysiology. As TACC3 was first identified in a screen for interactors of the hypoxia-responsive ARNT (aryl hydrocarbon receptor nuclear translator) protein (Sadek et al., 2000), and it has since been shown that TACC3 coiled-coils are a necessary co-factor for HIF complex assembly (Guo et al., 2015; Fig. 2B), TACC3 levels likely affect the integrity of the hypoxic response. Because oxygen levels are tightly regulated during embryonic development, it is imperative (as in the case of altered bioenergetics due to LETM1 deficiency) to consider the effect that their dysregulated fluctuation may have on migration events, among other processes pivotal to development.

The multitude of ways that TACC3 impacts development—cell cycle regulation, microtubule dynamicity, organized migration, and possibly hypoxia—merit further investigation in the context of WHS. It is worth noting that paralogous proteins TACC1 and TACC2 have been implicated in certain carcinomas, and thus could also impact migration (Conte et al., 2003; Gangisetty et al., 2004; Kimura and Okano, 2005; Takayama et al., 2012). Recently, TACC1 and TACC2 were revealed to possess plus-tip-tracking activity and the ability to influence MT dynamics in a manner similar to TACC3 (Lucaj et al., 2015; Rutherford et al., 2016). Given the homology between members of the TACC family, future work must be done to investigate the specific mechanisms by which the TACC family regulates developmental processes, with a special emphasis on whether TACCs 1 and 2 can rescue any disease phenotypes associated with TACC3-defficient cases of WHS. As for the possibility that TACC3 may impact the WHS phenotype in a manner dependent on the dosages of other WHS genes, the identification of an FGFR3-TACC3 fusion protein in human cancer cells has provided some initial insight (Carneiro et al., 2015). The oncogenic capacity of this fusion suggests that the activities of these two proteins are related in a way that is physiologically relevant to cancer progression; thus, although not fused in normal development, varied gene dosage due to WHS deletions may have a significant impact on processes lying downstream of both TACC3 and FGFR3 regulation.

### 5.5. Summary and future investigations

In conclusion, WHS is a developmental disorder with well-defined genetic causes, yet characterizing its cell biological profile remains an important barrier which must be broken to encourage successful clinical advances in the future. This review has focused on a series of genes on chromosome 4 whose sequential order is conserved at a second loci (on chromosome 8) within the human genome, and we have examined the roles of their protein products as described in the literature as a means of elucidating a cell migration-based functional relationship. The neural crest, a multipotent, migratory stem cell population, has been highlighted as an excellent candidate for relaying the genotypic aberrations of WHS to the structural, phenotypic level. Future studies looking into the mechanistic roles of TACC3, LETM1, FGFR3, and WHSC1 may reveal an interrelatedness between the levels of epigenetic modifications, intracellular signaling, and cytoskeletal regulation in the progression of the WHS disorder. Clarifying these intersecting cell biological irregularities will be indispensable in designing preventative and interventional therapies in the future.

At the most basic and immediate level, it will be informative to perform multiple WHS-mimicking genetic manipulations in tandem: this will mean creating animal models which either lack or contain extra WHS candidate genes in various combinations. Certain mouse lines already exist which could be utilized as a jumping-off point. For instance, mice that are homozygous null for *Fgfr3*, *Letm1*, or *Tacc3* alone currently exist (Simon and Bergemann, 2008). Simon and Bergemann have already articulated the need for polygenic models of WHS on the basis of the transcription factor/co-factor relationship of many WHS-implicated proteins such as Tacc3 and Ctbp1, whose shared interaction with transcription factor FOG-1 implies a relationship at the genetic level (Garriga-Canut and Orkin, 2004; Katz et al., 2002; Simon and Bergemann, 2008). In order to specifically interrogate motility-based defects in WHS, multigenic mouse models with mutations targeting the proteins reviewed here should be pursued. This represents an addendum to the work suggested by Simon and Bergemann, and a call for a more nuanced interrogation of how key cell biological processes such as signaling pathways and protein-protein interactions contribute to the WHS phenotype.

An equally important benchmark for the WHS field to push towards in the coming years will be the diversification of model systems used to study the disorder. While mouse models have facilitated the majority of our current insights into its pathology, and are particularly useful in that they allow for genotype-phenotype correlations which closely recapitulate the human disorder, no model has perfectly recreated the human phenotype (Fig. 3). Examining WHS candidate proteins in other systems may force us to consider significant details that have been overlooked at the cellular level. The *Xenopus* model system in particular has many merits as a tool to study development: the cranial neural crest and other neural cell types can easily be isolated and maintained in culture, allowing for a variety of migration and protein localization assays (DeSimone et al., 2005; Lowery et al., 2012; Milet and Monsoro-Burq, 2014). Moreover, facial cartilage forms in early tadpoles only a few days after oocyte fertilization, thus its morphology *in vivo* can be examined with unparalleled time efficiency. Quantitative morphometric analysis methods, optimized for the tadpole craniofacial area, also exist (Kennedy and Dickinson, 2014). With a new and broader perspective on both the modeling of WHS and the possible intricacies of its cell biology, it will be possible to better understand how the disorder progresses and to move toward the ultimate goal of disrupting such mechanisms.

## Figures and Tables

**Table 1:** WHS candidate genes. For each gene, the potentially WHS-related functions of its protein product are summarized, and relevant references detailing genetic, biochemical, and clinical data related to the gene are listed.

Gene	Function	References
*TACC3*	Transforming acidic coiled-coil protein: microtubule plus-end tracker, co-factor of the hypoxia inducible factor complex, regulator of epithelial-to-mesenchymal transition and cell migratory behaviors.	Gergely et al., 2000; Groisman et al., 2000; Peset and Vernos, 2008; Simon and Bergemann, 2008; Cyr et al., 2011; Zollino et al., 2014; Nwagbara et al., 2014.
*FGFR3*	Fibroblast growth factor receptor: involved in neural crest cell chemotaxis, regulator of cell proliferation, cell survival-promoting roles in neural development.	Colvin et al., 1996; Deng et al., 1996; Saarimäki-Vire et al., 2007; Puligilla et al., 2007; Simon and Bergemann, 2008; Katoh and Nakagama, 2014; Sato et al., 2011; Eswarakumar et al., 2005.
*LETM1*	Leucine-zipper and EF-hand-containing transmembrane protein: involved in mitochondrial bioenergetics, putative roles in regulating the cell cycle and cellular calcium homeostasis.	Endele et al., 1999; Zollino et al., 2003; Nowikovsky et al., 2004; Schlickum et al., 2004; Jiang et al., 2013; Doonan et al., 2014a, 2014b.
*WHSC1*	Histone H3 lysine methyltransferase; epigenetically influences TWIST transcription, interacts with beta-catenin.	Stec et al., 1998; Toyokawa et al., 2011; Ezponda et al., 2013.
*WHSC2*	mRNA processing and cell cycle regulation.	Wright et al., 1999.
